# Improved diagnostic sensitivity of human strongyloidiasis using point-of-care mixed recombinant antigen-based immunochromatography[Fn FN1]

**DOI:** 10.1051/parasite/2023063

**Published:** 2023-12-13

**Authors:** Patcharaporn Boonroumkaew, Lakkhana Sadaow, Penchom Janwan, Rutchanee Rodpai, Oranuch Sanpool, Punyisa Buadee, Chanida Suprom, Tongjit Thanchomnang, Pewpan M. Intapan, Wanchai Maleewong

**Affiliations:** 1 Department of Parasitology, Faculty of Medicine, Khon Kaen University 40002 Khon Kaen Thailand; 2 Mekong Health Science Research Institute, Khon Kaen University 40002 Khon Kaen Thailand; 3 Department of Medical Technology, School of Allied Health Sciences, Walailak University 80161 Nakhon Si Thammarat Thailand; 4 Hematology and Transfusion Science Research Center, Walailak University 80161 Nakhon Si Thammarat Thailand; 5 Faculty of Medicine, Mahasarakham University 44000 Maha Sarakham Thailand

**Keywords:** Strongyloidiasis, Neglected tropical disease, Recombinant antigen, Point-of-care, Serodiagnosis, Immunochromatography test

## Abstract

Strongyloidiasis is a neglected tropical disease that can cause fatal complications due to hyperinfection and disseminated strongyloidiasis in immunocompromised patients. We used two *Strongyloides stercoralis* recombinant antigenic proteins, L3NieAg.01 (NIE) and IgG-immunoreactive antigen (SsIR), to develop the recombinant antigen-based immunochromatography test (ICT) kit. We constructed and compared kits using either the NIE (NIE ICT kit) or the SsIR (SsIR ICT kit) antigens and a kit using a mixture of both (NIE-SsIR ICT kit) for detection of anti-*Strongyloides* IgG antibody in human serum samples. Serum samples from normal healthy individuals (Group I, *n* = 40), proven strongyloidiasis patients (Group II, *n* = 100), and those with other parasitic infections (Group III, *n* = 154) were evaluated. Sensitivity and specificity were 81.0% and 84.0% for the NIE ICT kit, 89.0% and 83.5% for the SsIR ICT kit, and 95.0% and 90.2% for the NIE-SsIR ICT kit, respectively. The NIE-SsIR ICT kit provided the best diagnostic results; it can supplement stool examination for clinical diagnosis and can be used to screen for asymptomatic *S. stercoralis* infection in people at risk in endemic areas. The NIE-SsIR ICT kit can also be used in large-scale sero-epidemiological investigations in endemic areas without the need for additional facilities or ancillary supplies.

## Abstract (fr)

La strongyloïdose est une maladie tropicale négligée qui peut entraîner des complications mortelles dues à une hyperinfection et à une strongyloïdose disséminée chez les patients immunodéprimés. Nous avons utilisé deux protéines antigéniques recombinantes de *Strongyloides stercoralis*, L3NieAg.01 (NIE) et l’antigène immunoréactif IgG (SsIR), pour développer un kit de test d’immunochromatographie (TIC) basé sur les antigènes recombinants. Nous avons construit et comparé des kits utilisant les antigènes NIE (kit NIE ICT), SsIR (kit SsIR ICT) ou un mélange des deux (kit NIE-SsIR ICT) pour la détection des anticorps IgG anti-S*trongyloides* dans des échantillons de sérum humain. Des échantillons de sérum provenant d’individus normaux en bonne santé (groupe I, n = 40), de patients atteints d’anguillulose avérée (groupe II, n = 100) et de patients atteints d’autres infections parasitaires (groupe III, n = 154) ont été évalués. La sensibilité et la spécificité étaient respectivement de 81,0 % et 84,0 % pour le kit NIE ICT, 89,0 % et 83,5 % pour le kit SsIR ICT, et 95,0 % et 90,2 % pour le kit NIE-SsIR ICT. Le kit NIE-SsIR ICT a fourni les meilleurs résultats de diagnostic ; il peut compléter l’examen des selles pour le diagnostic clinique et peut être utilisé pour dépister une infection asymptomatique à S. stercoralis chez les personnes à risque dans les zones d’endémie. Le kit NIE-SsIR ICT peut également être utilisé dans des enquêtes séro-épidémiologiques à grande échelle dans les zones endémiques sans nécessiter d’installations supplémentaires ou de fournitures auxiliaires.

## Introduction

Human strongyloidiasis is a neglected tropical disease estimated to infect 613.9 million people around the world [7]. The disease is caused by the nematodes *Strongyloides stercoralis* and *Strongyloides fuelleborni*. Although the former is mainly found in tropical and subtropical regions, it can be transmitted wherever faecal contamination occurs, such as in refugee camps with poor sanitation, or among those whose occupations expose them to soil contact [22, 24]. Strongyloidiasis may be uncomplicated or complicated. Persons with uncomplicated strongyloidiasis are asymptomatic or experience minor cutaneous and/or abdominal symptoms. Complicated strongyloidiasis can be fatal, especially in patients who are immunocompromised and/or receive immunosuppressants after solid-organ transplantation or corticosteroid therapy or cytotoxic treatments [22]. To prevent serious complications, accurate diagnosis is important [21]. Standard parasitological diagnostic methods detect *S. stercoralis* infection using concentration techniques and/or stool culture methods [29]. However, these techniques are tedious and time-consuming and require experienced parasitologists [29]. Appearance of larvae in faeces may fluctuate substantially over time, creating further difficulties for parasitological diagnosis [22]. Enzyme-linked immunosorbent assay (ELISA)-based methods have been used to detect *S. stercoralis* coproantigen from faecal specimens and have been suggested as a sensitive, simple and reliable approach. However, this method needs samples that produce strong and very unpleasant smells [13, 42]. Molecular technology has provided tools with high sensitivity and specificity, *i.e.*, conventional polymerase chain reaction (PCR) [21], nested-PCR [39], multiplex-PCR [36], quantitative real-time PCR [19, 39, 46], loop-mediated isothermal amplification [48], and droplet digital PCR [16]. Nevertheless, these tools need costly reagents and specialized facilities, confining their use to well-equipped laboratories [3].

Antibody-detection methods are widely used for serodiagnosis of human strongyloidiasis [2, 22]. Such methods are less demanding in terms of technical expertise than parasitological methods and require easily collected specimens (sera versus fresh stools) [45]. Antigens that are used include *Strongyloides* larval extract and recombinant antigens. They have been exploited in formats such as ELISA [2, 31, 43], western blot [10], indirect immunofluorescence assay [6], and luciferase immunoprecipitation system (LIPS) [30].

Two recombinant *Strongyloides* antigens (NIE (accession number AAD46493; [31] and SsIR (accession number AAB97359; [30]) are widely used for serodiagnosis of human strongyloidiasis and epidemiological studies of strongyloidiasis. A mixture of the two antigens (NIE and SsIR) has proved to be highly sensitive and specific using ELISA [43], biplex western blot [10], and LIPS [30].

Recently, a point of care test, the immunochromatography test (ICT) based on lateral flow assay, has been developed for antibody detection in human parasitoses. This format is a rapid, sensitive, and specific tool without the need for sophisticated equipment and can be used at the bedside [11, 14, 18, 32]. ICT tests for human strongyloidiasis have been developed [4, 28, 34]. To improve diagnostic values, in the present study, we used a mixture of the recombinant NIE and SsIR antigens combined with the ICT format for detection of total serum IgG antibodies in human strongyloidiasis. The results were compared with tests using either the NIE or the SsIR antigens individually.

## Materials and methods

### Study design

**Figure 1:**
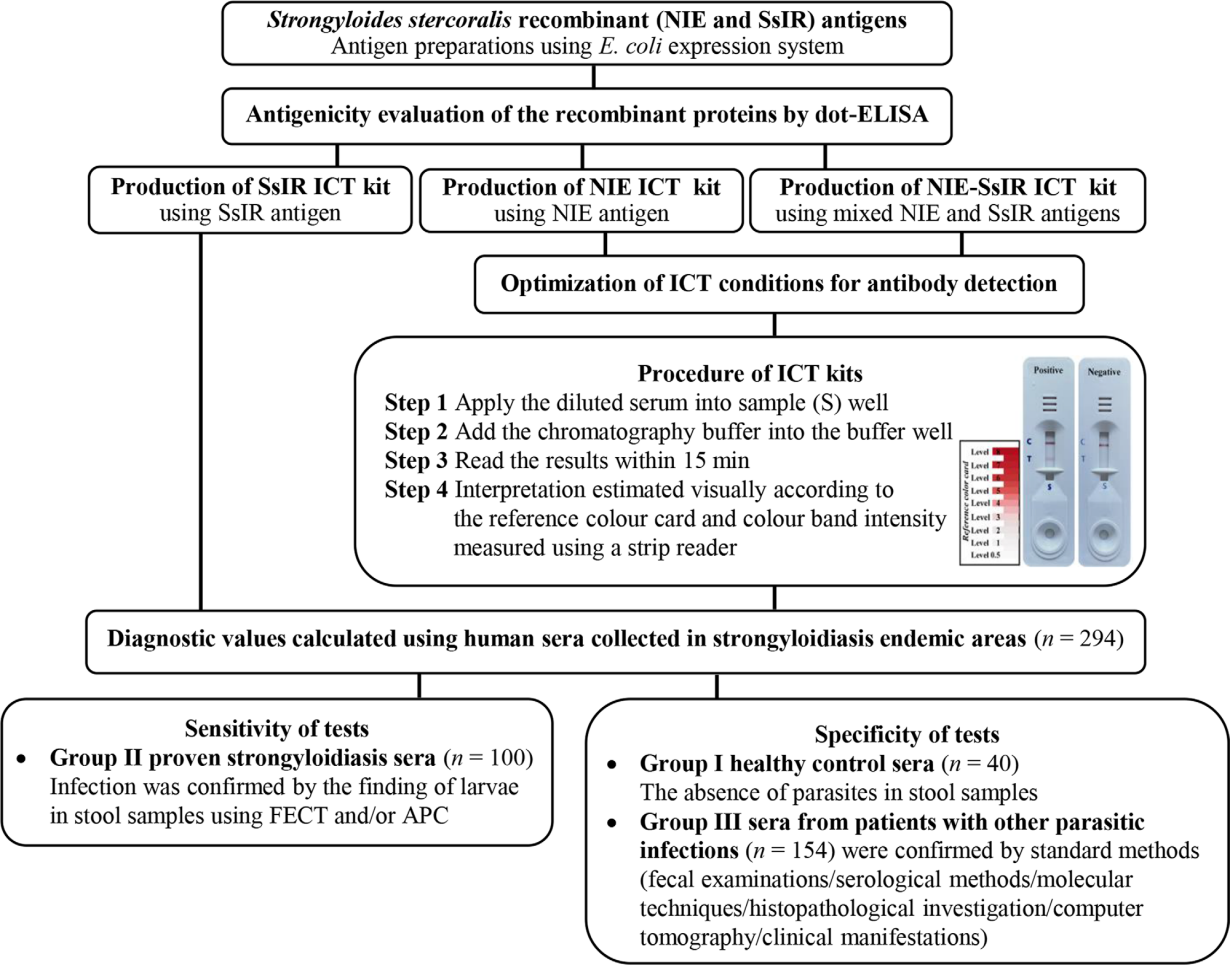
Flow diagram of study design for the NIE, SsIR, and NIE-SsIR ICT kits. The SsIR ICT kit procedure was performed as previously described by Boonroumkaew *et al.* [4].

All the serum samples were leftover specimens acquired from the serum bank at Khon Kaen University Faculty of Medicine and stored at −70 °C. The research was approved by the Human Ethics Committee at Khon Kaen University (HE611507). All data were fully anonymized, and the Human Ethics Committee waived the requirement for informed consent. The study design is shown in Figure 1.

### Human serum samples

The 294 serum samples available to us were separated into three groups: (i) control serum samples (*n* = 40) obtained from normal healthy individuals who were free from any intestinal protozoan or helminth infection at the time of blood collection (confirmed by the qualitative formalin ethyl-acetate concentration (FECT) technique) [12] and had a history of shoe wearing and non-exposure to soil occupation (Group I), (ii) serum samples from 100 patients in whom strongyloidiasis had been parasitologically confirmed using FECT and/or the agar-plate culture (APC) method [23] (Group II), and (iii) serum samples from 154 patients infected with other parasites (Group III). Parasitoses included in Group III included giardiasis (*n* = 7), amoebiasis (*n* = 10), blastocystosis (*n* = 10), opisthorchiasis (*n* = 10), taeniasis (*n* = 10), hookworm infections (*n* = 10), ascariasis (*n* = 10), trichuriasis (*n* = 10), and capillariasis (*n* = 10): all had been confirmed by parasitological examination of stool samples [12]. Cases of fascioliasis (*n* = 10) were confirmed by serological methods and clinical manifestations [25]. Cases of paragonimiasis (*n* = 10) were confirmed by the presence of eggs in sputa or faeces and by western blot [49]. Cysticercosis cases (*n* = 10) were confirmed by serology and CT [17]. Sparganosis cases (*n* = 7) were confirmed using histopathological investigation and PCR [5]. Trichinellosis cases (*n* = 10) were confirmed by using the ELISA technique [26]. Cases of angiostrongyliasis (*n* = 10) were confirmed by detection of eosinophilic meningitis, clinical suspicion of angiostrongyliasis, and serology using ELISA and ICT kits [40]. Gnathostomiasis cases (*n* = 10) were confirmed by serological methods with clinical manifestations and history of dietary preferences, as previously described [20]. Pooled positive and negative control sera were prepared by mixing equal volumes of sera from seven strongyloidiasis patients and seven healthy volunteers, respectively. These pooled positive and negative reference sera were further used as control sera for determination of within-day and between-day precision of the ICT kits. This study was performed according to the criteria of the STARD 2015 list for reporting diagnostic accuracy (Supplementary material 1) [9].

### *Strongyloides stercoralis* recombinant antigens

The *Strongyloides stercoralis* L3NieAg.01 (NIE) sequence (1–621 bp) deposited in the GenBank database (AF136445) was codon-optimized to be suitable for an *Escherichia coli* expression system and constructed into the pET22.b (+) vector (GenScript, Piscataway, NJ, USA). The recombinant NIE construct was transformed into a cloning host (*E. coli* JM109) (Novagen, Darmstadt, Germany) and an expression host (*E. coli* Rosetta-gami 2 (DE3)) (Novagen). The C-terminal-fused His-tagged NIE was inoculated into LB broth and induced with 1 mM IPTG and incubated at 37 °C overnight. Bacterial cells harvested from the culture were centrifuged and then resuspended in a lysis buffer with lysozyme. The suspension was then sonicated on ice. The recombinant NIE protein was initially in insoluble form and was dissolved in 8 M urea, pH = 8 (Elago Enterprises Pty Ltd., Cherrybrook, NSW, Australia).

The solubilized NIE protein fused with 6× His-tags at the C-terminal was purified by affinity chromatography using HisTrap™ HP 1 mL columns in the ÄKTA start protein purification system as recommended by the manufacturer (Global Life Sciences Solutions USA LLC, Marlborough, MA, USA). The purified NIE protein was examined on a 12% SDS–PAGE, and the protein concentration was measured using the Bradford Assay (Bio-Rad Laboratories, Inc., Hercules, CA, USA) with bovine serum albumin as a standard.

The *Strongyloides* recombinant IgG-immunoreactive (SsIR) antigen was prepared as reported previously [4]. The purified SsIR protein was examined on a 10% SDS–PAGE. Each recombinant antigen was stored at 4 °C before use.

### Evaluation of the antigenicity of the recombinant proteins by dot-ELISA

To confirm the antigenicity of the recombinant proteins, dot-ELISA was used. Antigens were dotted onto strips of a nitrocellulose (NC) membrane and incubated with the tested human serum sample, followed by a peroxidase-conjugated second antibody (slightly modified from Saenseeha *et al.* [35]). The 0.2 μm NC membrane (Bio-Rad Laboratories, Inc., Feldkirchen, Germany), was cut into 15 × 4 mm strips. The NIE antigen (2 μg in 1 μL) diluted in 6 M urea, the SsIR antigen (1 μg in 1 μL) diluted in distilled water, and the mixed NIE and SsIR antigens (1 μg each in 1 μL) diluted in 4.5 M urea were spotted separately onto an NC membrane (optimum antigen concentrations determined by checkerboard titration), air-dried for 15 min at room temperature (RT), and incubated at 37 °C for 2 h. To block the empty sites, the NC membrane was incubated for 30 min in 3% skimmed milk in 0.01 M PBS pH 7.5, with 0.1% Tween-20 (PBST). The NC membrane strips were washed five times with 1% skimmed milk in PBST and kept at −20 °C until use. Each strip was incubated for 30 min at RT with pooled positive and negative control sera (diluted 1:100 in 1% skimmed milk in PBS). To reduce nonspecific background from anti-bacterial antibodies in human sera, each serum sample was absorbed with *E. coli* lysate (1:100) at 37 °C for 30 min before reaction with the antigens. The strips with spotted antigens were washed five times with 1% skimmed milk in PBST before being incubated for 1 h at RT with goat anti-human IgG (H + L) HRP conjugate (ZyMAX, Invitrogen, Carlsbad, CA, USA) at a dilution of 1:20,000 (in 1% skimmed milk in PBS). After a further 5 washes with 1% skimmed milk in PBST, the dots were made visible using 3, 3′-diaminobenzidine-tetrahydrochloride solution with 30% hydrogen peroxide. After 5 min, the reaction was stopped by washing the strips with tap water. The strips were dried on filter paper. A brown dot indicated the presence of anti-*Strongyloides* antibody and was considered as a positive result. The absence of brown dots on the NC strips indicates that no anti-*Strongyloides* antibodies were present – a negative result.

### The construction of the NIE ICT kit

The NIE was incorporated into the “NIE ICT kit” used to detect total IgG antibody. The elements of the kit were as follows: sample pad (Kestrel BioSciences Co., Pathumthani, Thailand), conjugate release pad (glass microfiber filter GF33; Whatman Schleicher & Schuell, Dassel, Germany), nitrocellulose membrane (Sartorius Stedim Biotech SA, Göttingen, Germany) on which were sprayed the test (T) and control (C) lines, adsorbent pad (Kestrel BioSciences Co.), backing material (Kestrel BioSciences Co.) and cassette (Kestrel BioSciences Co.). The T line consisted of 2 mg/mL NIE in 6 mM urea and the C line consisted of goat anti-mouse IgG (1 mg/mL; 0.1 μL/mm) (Lampire Biological Laboratories, Pipersville, PA, USA). The conjugate release pad was sprayed with colloidal gold-conjugated mouse monoclonal anti-human IgG (Kestrel BioSciences Co.). The spraying procedure was done using an XYZ3210 Dispense Platform (BioDot, Irvine, CA, USA). The strip components were usually fixed in the backing material. The completed kit was placed in a resealable bag with a desiccant for storage.

**Figure 2:**
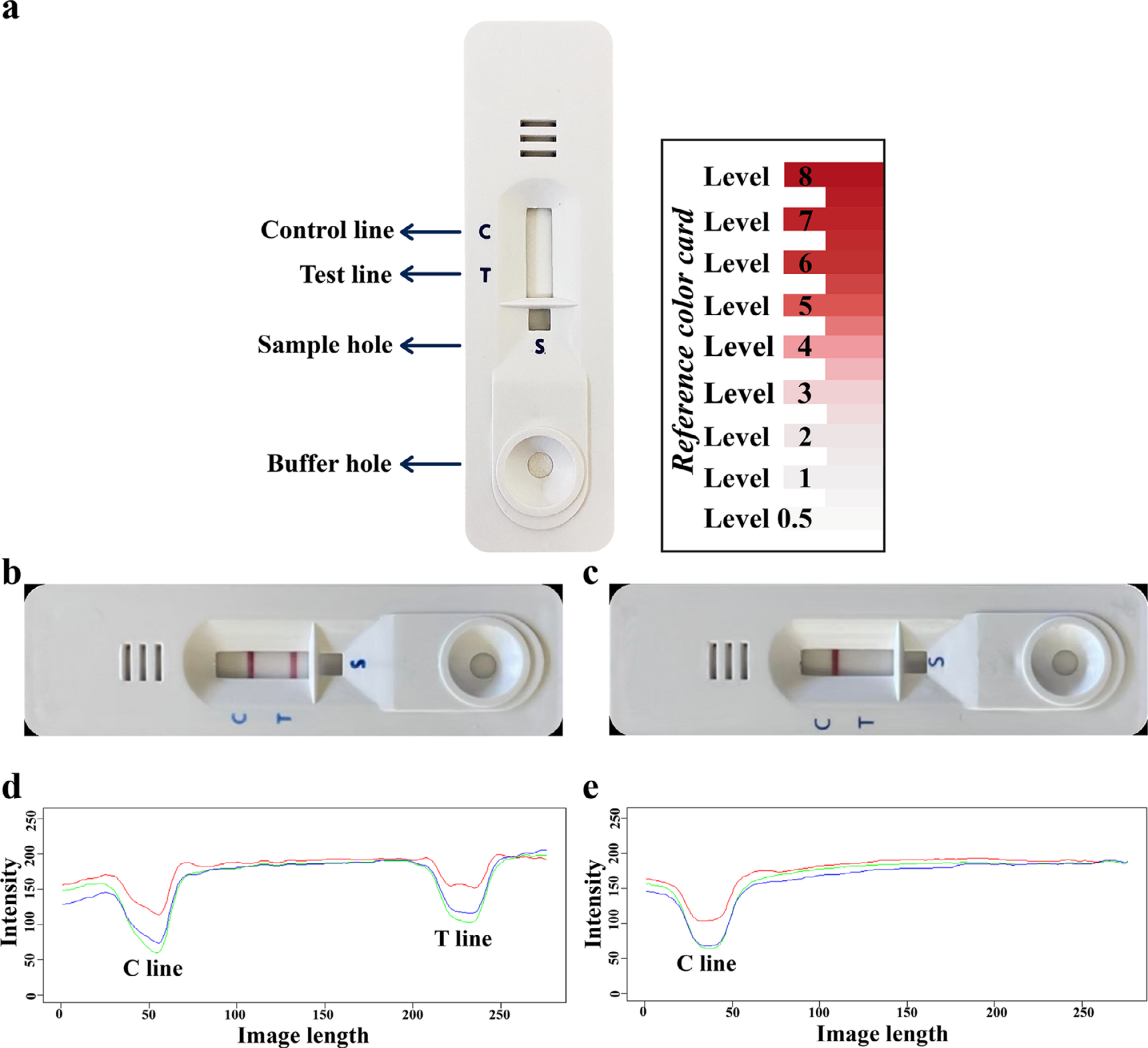
The parts of an ICT kit (left) and reference colour card (right) (a). An ICT kit showing a positive result (b) with a band at both control (C) and test (T) lines. An ICT kit showing a negative result (c) with a band only at the C line. The intensity value of the colour image is specified by the red, green, and blue parameters as separate integers from 0 to 255 with the 8-bit representation of a pixel in the image. The intensity values were plotted for C and T lines of the positive (d) and negative (e) ICT kits. The intensity values (d and e) were related to colour band intensity of C and T lines in the strips. S indicates sample hole.

To use the NIE ICT kit, the serum sample was mixed (1:100) with buffer, 10 μL of diluted serum was added into the S hole, and 120 μL of chromatography buffer into the buffer hole (Fig. 2a). Interpretation was solely based on the appearance of one or two red bands after 15 min. The intensity of any positive band (T line) was estimated visually by comparison with the reference colour card (the minimum cut-off level was 1) (Fig. 2a). Intensity of the colour band was also evaluated using an in-house strip reader (the intensity cut-off value < 164).

### The construction of the SsIR ICT kit

For kit production, the SsIR antigen [4] was used for IgG-antibody detection. The positive band intensity (T line) was estimated visually by comparison with the reference colour card (the minimum cut-off level was 1) (Fig. 2a). In addition, the intensity of the colour band was also evaluated using an in-house strip reader (the intensity cut-off value < 164).

### The construction of the NIE-SsIR ICT kit

The mixture of NIE and SsIR antigens were used to produce the “NIE-SsIR ICT kit” based on an approach similar to that used for the NIE ICT kit except that the T line was sprayed using a mixture of NIE and SsIR (1 mg/mL of each; volume ratio = 1:1) in 4.5 mM urea. To use the NIE-SsIR ICT kit, the serum sample was mixed (1:50) with buffer and 5 μL of this was added into the S hole, and 100 μL of chromatography buffer was applied into the buffer hole (Fig. 2a). Result interpretation was solely based on the appearance of the red band at 15 min; the intensity of any positive band at the T line was estimated visually (unaided) according to the reference colour card (the minimum cut-off level was > 0.5) (Fig. 2a). Intensity of the colour band was also evaluated using an in-house strip reader (the intensity cut-off value was < 167).

### Band intensity measurement

Colour image acquisition used the light reflection-based method with an in-house constructed strip reader. The intensity value of the colour image was specified by the red, green, and blue parameters as separate integers from 0 to 255 with the 8-bit representation of a pixel in the image. The image was transformed to grayscale using green colour intensity. The green colour represents the maximum intensity change between the T line and the background (Figs. 2b–2e).

We evaluated the different colour intensity values at the T line region by comparison with the reference colour card. We analysed the pixel data using linear modelling in R (https://cran.r-project.org/). A regression model was used to determine a pixel intensity to band level. A standard model was created based on both pixel intensity and expert comparison with the colour reference card. This model was evaluated based on a linear relationship between data training and level. Equation (1) describes the linear model between pixel intensity and reference card level. Equations (2) and (3) were used to calculate model parameters (model slope and intercept):

(1) y=mx+c

where: *y* = level, *m* = slope, *x* = pixel intensity and *c* = intercept

(2) $$m=\frac{\sum_{i=1}^{n}\left(x_{i}-\bar{x}\right)\left(y_{i}-\bar{y}\right)}{\sum_{i=1}^{n}{\left(x_{i}-\bar{x}\right)}^{2}}$$

where: *n* = number of observations, *x*_*i*_ = training pixel intensity and *y*_*i*_ = training output

(3) $$c=\bar{y}-m\bar{x}$$

### Diagnostic performance of NIE, SsIR and NIE-SsIR ICT kits

The diagnostic parameters (sensitivity, specificity, and positive and negative likelihood ratios) [15] for each ICT kit were calculated based on tests of the three groups of sera (normal healthy sera (Group I), human strongyloidiasis sera (Group II), and sera from patients with other parasitic infections (Group III)). Stata 10 software was used for the calculations [41].

## Results

### Protein expression

**Figure 3:**
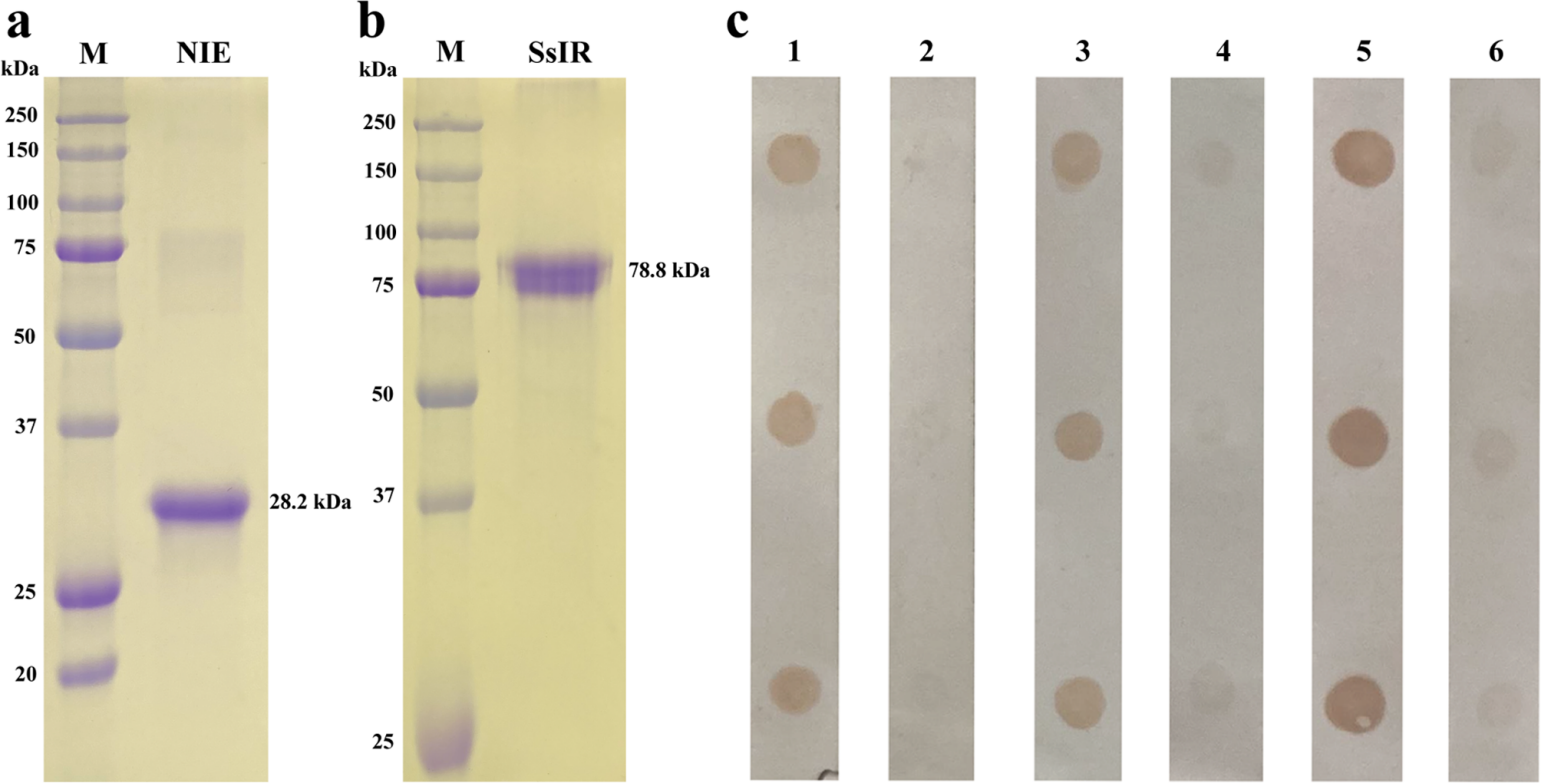
The purified NIE (a) and SsIR (b) fusion-tagged proteins visualized following electrophoresis through 12% and 10% SDS–PAGE, respectively. The gels were stained with Coomassie Brilliant Blue. Representative dot ELISAs (c) using NIE (1–2), SsIR (3–4), and mixed NIE and SsIR (5–6) proteins as the antigens probed with pooled positive (1, 3, 5) and negative (2, 4, 6) sera. M indicates molecular mass maker.

NIE and SsIR proteins were successfully expressed in the *E. coli* system and subsequently purified. The NIE protein has a molecular mass of 28.2 kDa (including the His-tag) (Fig. 3a) and the SsIR protein has a molecular mass of 78.8 kDa (including Nus•Tag, His-Tag (N and C term), and S•Tag) (Fig. 3b). Human antigenicity of both recombinant proteins was shown by dot-ELISA (Fig. 3c).

### The ICT kits

**Figure 4:**
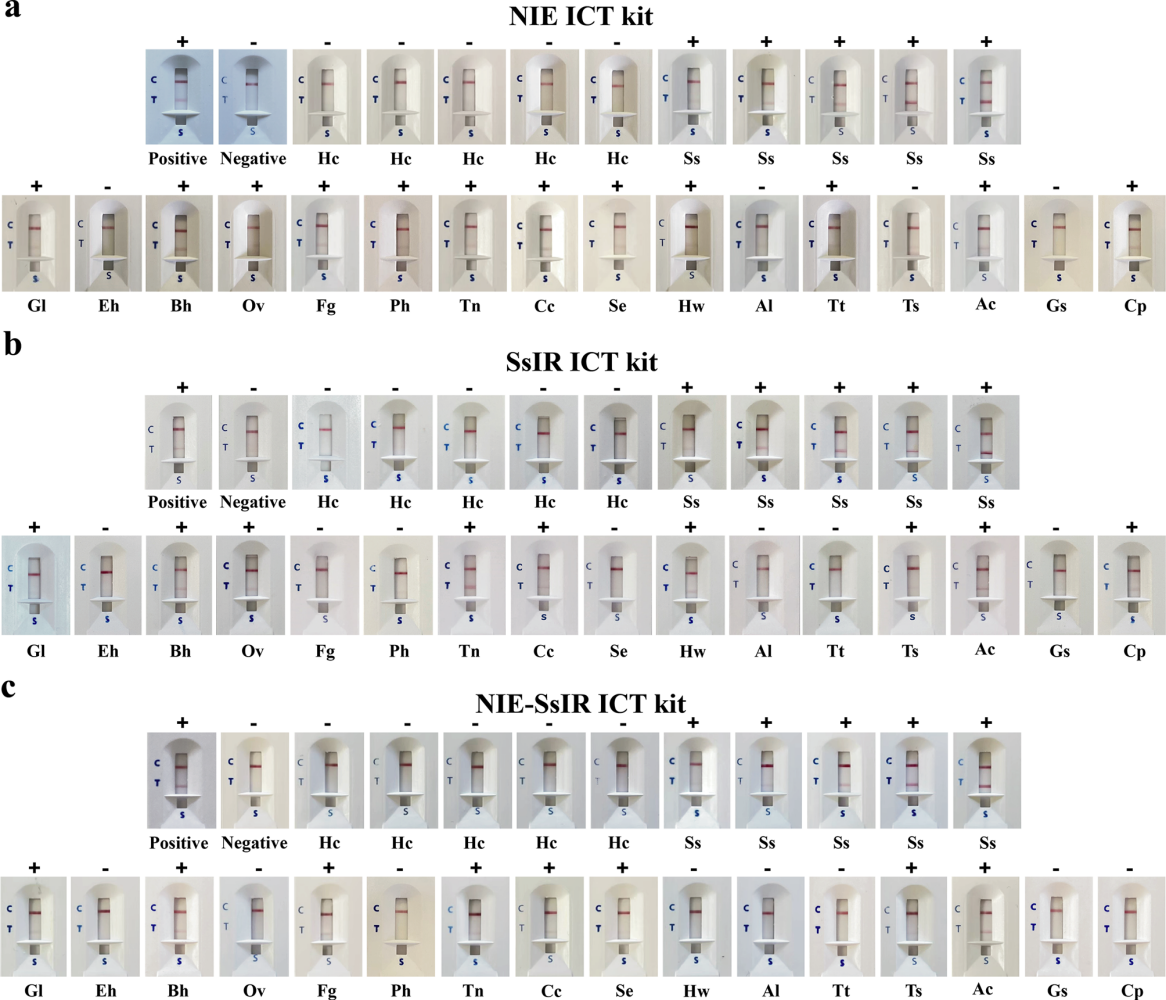
Representative results of the NIE, SsIR, and NIE-SsIR ICT kits, Positive, positive pooled serum samples; Negative, negative pooled serum samples; Hc, healthy control; Ss, proven strongyloidiasis; Gl, giardiasis; Eh, amoebiasis; Bh, blastocystosis; Ov, opisthorchiasis; Fg, fascioliasis; Ph, paragonimiasis; Tn, taeniasis; Cc, cysticercosis; Se, sparganosis; Hw, hookworm infections; Al, ascariasis; Tt, trichuriasis; Ts, trichinellosis; Ac, angiostrongyliasis; Gs, gnathostomiasis; Cp, capillariasis. The intensity cut-off levels for a positive result of the NIE, SsIR, and NIE-SsIR ICT kits were 1, 1, and >0.5, respectively by the naked eye and <164, <164, and <167, respectively by the in-house strip reader. The “+” and “−” symbols indicated positive and negative results.

**Figure 5:**
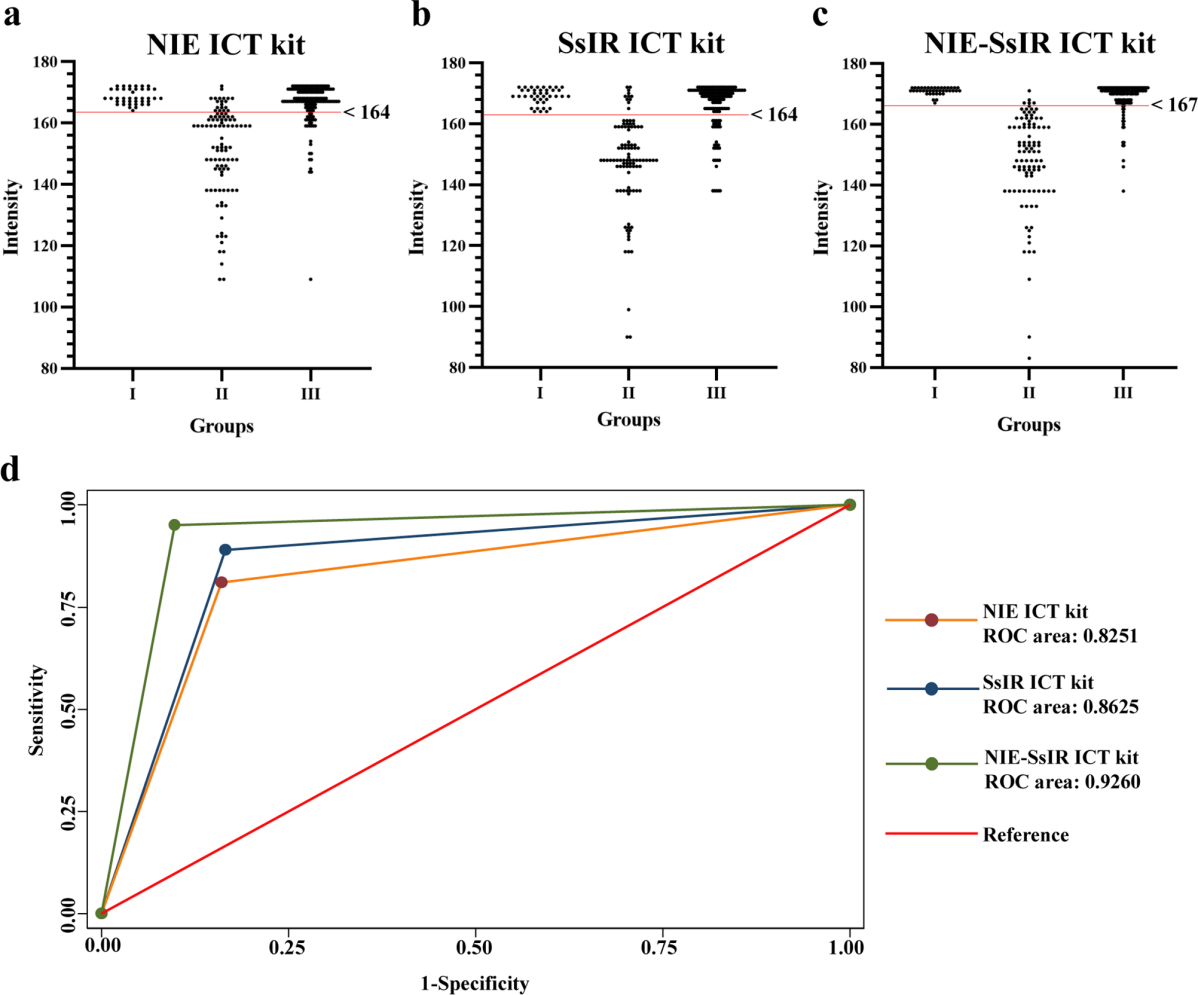
The intensity values of NIE (a), SsIR (b), and NIE-SsIR (c) ICT kits were evaluated using an in-house strip reader (red line indicates cut-off intensity value). Groups I, II, and III represented healthy controls, proven strongyloidiasis, and other parasitic infections, respectively. Any value above the cut-off value (red horizontal lines) is negative. Receiver operator characteristic (ROC) area analyses of the NIE, SsIR, and NIE-SsIR ICT kits to compare accuracy of all kits with gold standard methods (d).

The NIE, SsIR, and NIE-SsIR ICT kits results were read by observing the coloured bands visible to the naked eye in the window of the test cassette (Fig. 4) and were evaluated using an in-house strip reader (Figs. 5a–5c) (Supplementary material 2). Correlation results were found when interpretations by the naked eye and the in-house strip reader. If coloured bands appeared at both the C and T lines, the result was positive. Conversely, a coloured band at the C line alone denoted that the sample was negative. If no band appeared at the C line, then the assay was invalid (faulty kit) and should be repeated with a new kit. Between-day precision was determined by performing each test on the same pooled positive and negative reference sera on different days; no day-to-day variation was seen. To compare the accuracy of all ICT kits with the gold-standard methods (see human serum samples above), we used the receiver operator characteristic (ROC) area under the curve (Fig. 5d).

### Diagnostic values of the NIE ICT kit

The NIE ICT kit was positive for human strongyloidiasis in 81 of 100 proven cases, with 81.0% (95%CI [71.9–88.2]) sensitivity, while the kit was negative for all normal healthy and 123 out of 154 sera from patients with other parasitic infections, with 84.0% (163/194) (95%CI [78.1–88.9]) specificity. Cross-reactions were seen in 31 cases (Table 1). The positive likelihood ratio was 5.07 (95%CI [3.62–7.10]) and the negative likelihood ratio was 0.23 (95%CI [0.15–0.34]).

**Table 1:** Results of recombinant antigen-based immunochromatography tests (NIE, SsIR, and NIE-SsIR ICT kits) for detection of anti-*Strongyloides* IgG antibody in human serum samples.

Conditions represented by sera	No. of sera tested	NIE ICT kit	SsIR ICT kit	NIE-SsIR ICT kit
		No. positive^2^	No. positive^2^	No. positive^2^
Group I				
Healthy controls	40	0	0	0
Group II				
Proven strongyloidiasis	100	81	89	95
Group III				
Giardiasis	7	1	2	2
Amoebiasis	10	1	3	0
Blastocystosis	10	2	4	3
Opisthorchiasis	10	5	2	0
Fascioliasis	10	3	1	3
Paragonimiasis	10	2	1	0
Taeniasis	10	2	3	1
Cysticercosis	10	3	2	2
Sparganosis	7	3	2	3
Hookworm infections	10	4	4	1
Ascariasis	10	0	0	0
Trichuriasis	10	2	0	0
Trichinellosis	10	0	2	1
Angiostrongyliasis	10	1	3	2
Gnathostomiasis	10	0	2	0
Capillariasis	10	2	1	1
			Diagnostic values [95% CI^1^]	
Sensitivity (%)		81.0 [71.9–88.2]	89.0 [81.2–94.4]	95.0 [88.7–98.4]
Specificity (%)		84.0 [78.1–88.9]	83.5 [77.5–88.4]	90.2 [85.1–94.0]
Positive likelihood ratio		5.07 [3.62–7.10]	5.40 [3.90–7.46]	9.70 [6.31–14.90]
Negative likelihood ratio		0.23 [0.15–0.34]	0.13 [0.08–0.23]	0.06 [0.02–0.13]

1 CI, confidence interval.

2 The results were correlated between interpretations by the naked eye and the in-house strip reader.

### Diagnostic values of the SsIR ICT kit

The SsIR ICT kit was positive for human strongyloidiasis in 89 of 100 proven cases, with 89.0% (95%CI [81.2–94.4]) sensitivity, while it was negative for all normal healthy and 122 out of 154 other parasitic infections, with 83.5% (162/194) (95%CI [77.5–88.4]) specificity. In 32 serum samples, there were cross-reactions (Table 1). The positive and negative likelihood ratios were 5.40 (95%CI [3.90–7.46]) and 0.13 (95%CI [0.08–0.23]), respectively.

### Diagnostic values of the NIE-SsIR ICT kit

The NIE-SsIR ICT kit was positive for human strongyloidiasis in 95 of 100 proven cases, with 95.0% (95%CI [88.7–98.4]) sensitivity, while it was negative for all normal healthy and 135 out of 154 other parasitic infections, with 90.2% (175/195) (95%CI [85.1–94.0]) specificity. Nineteen serum samples yielded cross-reactions (Table 1). The positive and negative likelihood ratios were 9.70 (95%CI [6.31–14.90]) and 0.06 (95%CI [0.02–0.13]), respectively.

## Discussion

Previously, larval somatic antigens of either *Strongyloides ratti* [33] or *S. stercoralis* [34] were used to develop serodiagnostic tests for human strongyloidiasis. However, widespread use of these antigens is hampered by the very limited amount of antigenic material that can be extracted from native *Strongyloides* parasites and the length of time required to cultivate the parasite in the laboratory. On the other hand, antigens based on recombinant proteins can be mass-produced, by passing these limitations. Two types of recombinant antigen, NIE and SsIR, have been used for antibody detection, demonstrating good diagnostic values for human strongyloidiasis with high sensitivity (ranging from 81 to 100%) and specificity (ranging from 91 to 100%) using LIPS [30], western blot [10] and ELISA [30, 37, 43].

Recently, the “SsRapid^TM^” test, a lateral-flow rapid dipstick test using a mixture of recombinant NIE and Ss1a antigens for IgG4 antibody detection, has been used for the diagnosis of human strongyloidiasis in serum samples. The test showed diagnostic sensitivity of 91.3% (21/23) and specificity of 100% (88/88) when evaluated with sera from Malaysia [50] and sensitivity of 82.1% (69/84) and specificity of 96.0% (24/25) when evaluated with sera from Northeast Thailand [27]. ICT kits have also been developed as POC testing tools for diagnosis of strongyloidiasis [4, 28, 34]. Sadoaw *et al.* [34] developed “the strongyloidiasis ICT kit” using *S. stercoralis* larval soluble extract antigen for detection of IgG antibodies and showed diagnostic sensitivity of 93.3% (56/60) and specificity of 83.7% (87/104) when evaluated with sera from Thailand. Boonroumkaew *et al.* [4] developed the ICT kits for IgG and IgG4 antibody detections using the recombinant SsIR antigen. These had sensitivities of 91.7% (55/60) and 78.3% (47/60), as well as specificities of 83.8% (88/105) and 84.8% (89/105), respectively, when evaluated with sera from the same panel. Recently, Noordin *et al.* [28] developed the “Ss Rapid^®^”, a cassette rapid IgG4 test using recombinant NIE antigen and showed diagnostic specificity of 94.5% (208/220) and sensitivity of 96.9% (31/32) when evaluated with panels of sera from Malaysia, Iran and Thailand.

Use of mixed recombinant antigens for anti-*Strongyloides* antibody detection can improve the diagnostic sensitivity and specificity of tests beyond that seen using a single recombinant antigen [10, 30, 50]. To the authors’ knowledge, there have been no reports of any ICT kit using mixed NIE and SsIR antigens for detection of IgG antibody for serodiagnosis of human strongyloidiasis. In the present study, using a panel of human sera from Thailand, we evaluated the diagnostic values of the NIE-SsIR ICT kit and showed a sensitivity of 95.0% and specificity of 90.2%. This represents an improvement over the ICT kits using only the NIE antigen (81.0% sensitivity and 84.0% specificity) and only the SsIR antigen (89.0% sensitivity and 83.5% specificity). However, the sensitivity (95.0%; 95/100) and specificity (90.2%;175/194) of the NIE-SsIR ICT kit was slightly lower than the sensitivity (96.9%; 31/32) and specificity (94.5%; 208/220) previous reported for the “Ss Rapid^®^” kit [28]. The different sensitivities and specificities of the above serological tests will depend on details of each method, antigens, type of antibody detection, cut-off values, and tested panel of sera used [2]. Nevertheless, the NIE-SsIR ICT platform offers a novel approach for diagnosis of human strongyloidiasis. The finding that the NIE-SsIR ICT performs better than ICTs using single antigens only supports the perception that different epitopes may be antigenic to varying degrees among individuals [30, 44]. The NIE-SsIR ICT possibly detects more antigenic epitopes in the combined antigens, resulting in sensitivity increasing. For specificity evaluation, this condition may be the optimal concentrations of both antigens in the NIE-SsIR ICT kit.

Cross-reactivity (Table 1) with some sera from Group III (giardiasis, blastocystosis, hookworm infections) might be because some serum samples were collected from strongyloidiasis-endemic areas in northeast Thailand and those individuals may have had asymptomatic strongyloidiasis. All the kits showed cross-reactions with some serum samples from patients with a range of other parasitoses (Table 1). However, cross reactions do not cause a real problem in a clinical setting, because the clinical presentations of these parasitoses are distinct from those of strongyloidiasis. Another factor to consider when evaluating sensitivity is that some strongyloidiasis sera, in which *S. stercoralis* was found using FECT and the APC method, were negative according to our ICT kit (Table 1). This may have been because those serum samples were collected during the acute phase of strongyloidiasis [47], or from immunodeficient patients [38]. In either case, a low antibody response is to be expected. When regarding the calculated specificities of three kits based on control serum samples (Group I), the results all showed 100%. When the specificities were calculated based on the parasitoses group, Group III showed 79.9% (123/154) for the NIE ICT kit, 79.2% (122/154) for the SsIR ICT kit, and 87.7% (135/154) for the NIE-SsIR ICT kit, respectively. The promising tests were verified in laboratory settings and using an artificial set of samples. Clinicians who are working in this area need to be aware of the limitations of this study, and the methods need to be evaluated further in a real-world population.

In conclusion, the NIE-SsIR ICT kit showed higher sensitivity and specificity than the other kits. As a new diagnostic tool, the NIE-SsIR ICT kit returns a result quickly, is simple to use, and can supplement stool examination for clinical diagnosis of strongyloidiasis. The use of recombinant *S. stercoralis* antigens means that antigenic material can be mass-produced. This kit can be used at the local level in large-scale sero-epidemiological investigations in endemic areas without the need for additional facilities or ancillary supplies. Due to the unique autoinfection cycle exhibited by *Strongyloides*, lifelong latent infection can occur. Given increased global mobility, the CDC recommends that all individuals who have ever resided in *S. stercoralis*-endemic areas should be screened for asymptomatic *S. stercoralis* infection prior to administration of steroids [8], immunosuppressive agents, or chemotherapy [1]. Also, the test has the potential for commercial translation, up-scaling and making an impact on disease control. The ICT kit should be further developed for antibody detection in whole-blood samples and evaluated in the field for use with fingerstick blood samples.

## Supplementary information

The Supplementary materials of this article are available at https://www.parasite-journal.org/10.1051/parasite/2023063/olm. *Supplementary material 1.* STARD 2015 reporting guideline for diagnostic accuracy studies. *Supplementary material 2.* The Intensity of the colour band (T line) was visually estimated by comparison with the reference colour card and evaluated using an in-house strip reader.
