# Supplementary material for: Improved diagnostic sensitivity of human strongyloidiasis using point-of-care mixed recombinant antigen-based immunochromatography
Source: Parasite. 2023 Dec 13;30:60. doi: 10.1051/parasite/2023063 (PMC10723528; doi:10.1051/parasite/2023063)
Supplement: Supplementary file 1 — Supplementary material 1. STARD 2015 reporting guideline for diagnostic accuracy studies. Supplementary material 2. The Intensity of the colour band (T line) was visually estimated by comparison with the reference colour card and evaluated using an in-house strip reader. [file parasite-30-60-s1.zip › Supplementary material 2.pdf]

Supplementary material 2. **The Intensity of the color band (T line) was visually estimated by comparison with the reference color card and evaluated using an in-house strip reader.**

	**Results**						
	**NIE ICT kit**		**SsIR ICT kit**		**NIE-SsIR ICT kit**		
	**Reference color card**	**In-house strip reader**	**Reference color card**	**In-house strip reader**	**Reference color card**	**In-house strip reader**	
**Code**	**the minimum cutoff level was 1**	**the intensity cutoff value < 164**	**the minimum cutoff level was 1**	**the intensity cutoff value < 164**	**the minimum cutoff level was >0.5**	**the intensity cutoff value was < 167**	**Group**
**Hc-1**	N	172	N	171	N	172	I
**Hc-2**	N	168	>0.5	164	N	171	I
**Hc-3**	N	168	N	172	N	170	I
**Hc-4**	0.5	167	N	169	N	171	I
**Hc-5**	N	172	N	169	N	172	I
**Hc-6**	N	171	N	170	N	171	I
**Hc-7**	N	172	N	172	N	172	I
**Hc-8**	0.5	167	N	169	N	170	I
**Hc-9**	N	168	N	169	N	171	I
**Hc-10**	>0.5	165	N	171	N	171	I
**Hc-11**	N	168	N	169	N	172	I
**Hc-12**	N	171	N	168	0.5	167	I
**Hc-13**	N	172	N	172	N	172	I
**Hc-14**	>0.5	166	N	171	N	171	I
**Hc-15**	N	171	N	171	N	171	I
**Hc-16**	>0.5	166	N	172	N	170	I
**Hc-17**	0.5	167	N	170	N	172	I
**Hc-18**	N	170	N	171	N	170	I
**Hc-19**	N	168	0.5	167	N	172	I
**Hc-20**	N	171	N	172	N	171	I
**Hc-21**	>0.5	164	>0.5	165	N	170	I
**Hc-22**	N	172	N	169	N	172	I
**Hc-23**	>0.5	166	>0.5	164	N	172	I
**Hc-24**	>0.5	166	N	172	N	172	I
**Hc-25**	N	171	N	168	N	171	I
**Hc-26**	N	171	N	172	N	171	I

**Hc-27**	>0.5	165	>0.5	165	N	168	I
**Hc-28**	N	171	N	169	N	171	I
**Hc-29**	0.5	167	0.5	167	N	172	I
**Hc-30**	0.5	167	N	169	N	171	I
**Hc-31**	N	168	>0.5	164	N	172	I
**Hc-32**	>0.5	166	N	168	N	171	I
**Hc-33**	N	168	>0.5	165	N	168	I
**Hc-34**	0.5	167	N	171	N	172	I
**Hc-35**	N	171	>0.5	165	N	171	I
**Hc-36**	N	168	N	172	N	171	I
**Hc-37**	N	168	N	171	N	170	I
**Hc-38**	N	172	N	170	N	172	I
**Hc-39**	N	168	N	169	N	171	I
**Hc-40**	>0.5	166	N	169	N	172	I
**Ss-1**	>2	148	N	172	3	146	II
**Ss-2**	>0.5	164	2	152	1	162	II
**Ss-3**	0.5	167	3	146	0.5	167	II
**Ss-4**	>3	138	>2	148	>3	138	II
**Ss-5**	>2	148	3	146	3	145	II
**Ss-6**	3	146	2	154	3	146	II
**Ss-7**	>1	159	>3	138	3	143	II
**Ss-8**	1	161	2	152	2	152	II
**Ss-9**	3	146	3	147	2	151	II
**Ss-10**	5	109	>2	148	3	146	II
**Ss-11**	>1	159	4	126	>2	148	II
**Ss-12**	>2	148	>2	148	2	154	II
**Ss-13**	>1	159	>1	159	>0.5	165	II
**Ss-14**	3	146	>2	148	1	160	II
**Ss-15**	>2	148	4	125	>3	138	II
**Ss-16**	4	123	>3	137	3	146	II
**Ss-17**	>2	148	4	124	4	123	II
**Ss-18**	>3	133	>4	118	4	126	II

**Ss-19**	>3	134	4	126	3	146	II
**Ss-20**	0.5	167	>2	148	2	153	II
**Ss-21**	2	151	>1	159	2	151	II
**Ss-22**	>3	138	4	123	3	145	II
**Ss-23**	3	145	>2	148	2	154	II
**Ss-24**	N	168	N	169	N	168	II
**Ss-25**	1	161	3	147	>2	148	II
**Ss-26**	>1	159	4	127	>3	138	II
**Ss-27**	>2	148	>2	148	2	153	II
**Ss-28**	1	162	1	160	1	162	II
**Ss-29**	>3	133	>3	138	>3	138	II
**Ss-30**	4	123	>1	159	2	154	II
**Ss-31**	N	171	N	171	N	171	II
**Ss-32**	2	152	1	161	1	162	II
**Ss-33**	3	145	>1	159	>0.5	165	II
**Ss-34**	0.5	167	0.5	167	0.5	167	II
**Ss-35**	1	163	>2	148	>3	138	II
**Ss-36**	>1	159	1	160	>0.5	165	II
**Ss-37**	3	145	1	161	>0.5	164	II
**Ss-38**	1	162	>4	118	>3	133	II
**Ss-39**	>1	159	>3	138	3	143	II
**Ss-40**	1	161	2	152	>1	159	II
**Ss-41**	>1	159	2	153	2	151	II
**Ss-42**	1	162	>2	148	1	162	II
**Ss-43**	2	152	>3	138	2	152	II
**Ss-44**	>3	138	>3	138	>2	148	II
**Ss-45**	1	162	3	147	4	125	II
**Ss-46**	1	163	6	90	4	121	II
**Ss-47**	4	124	N	170	2	153	II
**Ss-48**	3	145	>2	148	>1	159	II
**Ss-49**	1	162	3	146	3	146	II
**Ss-50**	>0.5	164	2	153	0.5	167	II

**Ss-51**	4	129	>4	118	>4	118	II
**Ss-52**	>4	118	2	152	2	151	II
**Ss-53**	>3	138	5	99	7	83	II
**Ss-54**	5	109	3	147	>3	138	II
**Ss-55**	1	161	3	146	3	145	II
**Ss-56**	N	168	>2	148	>3	138	II
**Ss-57**	>4	118	>3	138	4	126	II
**Ss-58**	1	161	2	152	3	146	II
**Ss-59**	2	152	2	153	>1	159	II
**Ss-60**	>0.5	165	3	146	3	144	II
**Ss-61**	>3	138	>1	159	2	156	II
**Ss-62**	1	160	3	146	>1	159	II
**Ss-63**	1	161	>2	148	2	151	II
**Ss-64**	>0.5	166	N	168	1	162	II
**Ss-65**	>0.5	164	1	161	2	154	II
**Ss-66**	4	123	>2	148	3	145	II
**Ss-67**	N	168	>1	159	2	153	II
**Ss-68**	>0.5	165	>1	159	>1	159	II
**Ss-69**	>1	159	2	152	2	153	II
**Ss-70**	N	168	>2	148	1	162	II
**Ss-71**	>0.5	164	N	169	1	163	II
**Ss-72**	>0.5	165	2	153	1	160	II
**Ss-73**	>2	148	N	172	1	161	II
**Ss-74**	>1	159	>0.5	165	>0.5	165	II
**Ss-75**	N	172	1	160	>0.5	166	II
**Ss-76**	2	151	>1	158	>0.5	164	II
**Ss-77**	1	163	2	152	1	160	II
**Ss-78**	2	151	>2	148	>1	159	II
**Ss-79**	>1	159	>3	138	>1	159	II
**Ss-80**	N	168	1	161	>0.5	164	II
**Ss-81**	>3	138	N	169	2	154	II
**Ss-82**	>3	138	>1	159	1	163	II

**Ss-83**	1	162	2	149	>3	138	II
**Ss-84**	2	153	4	125	>4	118	II
**Ss-85**	2	152	3	144	>3	138	II
**Ss-86**	>3	133	3	139	>2	148	II
**Ss-87**	>1	159	1	160	>1	159	II
**Ss-88**	>2	148	3	146	>3	133	II
**Ss-89**	1	161	4	126	5	109	II
**Ss-90**	>1	159	2	153	3	144	II
**Ss-91**	2	152	>3	138	3	145	II
**Ss-92**	N	168	N	168	>1	159	II
**Ss-93**	4	121	4	122	>4	118	II
**Ss-94**	>3	138	3	146	>3	138	II
**Ss-95**	3	144	>2	148	>2	148	II
**Ss-96**	2	155	>2	148	>3	133	II
**Ss-97**	5	114	6	90	6	90	II
**Ss-98**	>1	159	>2	148	>3	138	II
**Ss-99**	>1	159	>1	159	>2	148	II
**Ss-100**	3	143	2	150	>3	133	II
**Gl-1**	N	172	N	171	N	171	III
**Gl-2**	1	161	>2	148	1	160	III
**Gl-3**	0.5	167	2	152	>0.5	166	III
**Gl-4**	0.5	167	N	171	N	170	III
**Gl-5**	0.5	167	N	172	N	171	III
**Gl-6**	>0.5	164	N	170	N	172	III
**Gl-7**	>0.5	165	>0.5	165	N	170	III
**Eh-1**	N	171	N	171	N	171	III
**Eh-2**	N	168	>1	159	N	168	III
**Eh-3**	N	172	2	153	0.5	167	III
**Eh-4**	2	171	N	169	N	172	III
**Eh-5**	>0.5	166	N	171	N	171	III
**Eh-6**	N	170	N	171	N	170	III
**Eh-7**	N	171	N	172	N	172	III

**Eh-8**	N	172	N	170	N	171	III
**Eh-9**	N	168	N	171	N	170	III
**Eh-10**	N	172	2	152	N	172	III
**Bh-1**	N	171	N	171	N	171	III
**Bh-2**	N	168	>3	138	2	154	III
**Bh-3**	0.5	167	>2	148	2	153	III
**Bh-4**	0.5	167	N	171	N	172	III
**Bh-5**	>0.5	164	N	171	N	171	III
**Bh-6**	N	172	>0.5	164	N	168	III
**Bh-7**	>2	148	>1	159	2	153	III
**Bh-8**	N	168	N	172	N	172	III
**Bh-9**	2	154	2	154	0.5	167	III
**Bh-10**	N	172	N	171	N	172	III
**Ov-1**	1	163	0.5	167	N	171	III
**Ov-2**	1	162	N	170	N	172	III
**Ov-3**	1	161	N	172	N	170	III
**Ov-4**	1	160	N	171	N	172	III
**Ov-5**	N	171	N	171	N	171	III
**Ov-6**	0.5	167	2	152	N	172	III
**Ov-7**	1	161	>0.5	165	N	170	III
**Ov-8**	N	171	N	171	N	171	III
**Ov-9**	0.5	167	N	169	N	172	III
**Ov-10**	>0.5	166	>2	148	0.5	167	III
**Fg-1**	N	171	N	172	N	170	III
**Fg-2**	0.5	167	>0.5	165	N	168	III
**Fg-3**	>0.5	165	0.5	167	>0.5	165	III
**Fg-4**	5	109	>2	148	3	146	III
**Fg-5**	2	153	N	171	>1	159	III
**Fg-6**	N	172	N	171	N	172	III
**Fg-7**	1	162	N	168	N	171	III
**Fg-8**	N	172	>0.5	165	0.5	167	III
**Fg-9**	>0.5	164	N	169	N	172	III

**Fg-10**	0.5	167	N	171	N	171	III
**Ph-1**	>1	159	N	169	N	170	III
**Ph-2**	>0.5	166	N	171	N	171	III
**Ph-3**	N	171	N	172	N	172	III
**Ph-4**	N	170	N	170	N	170	III
**Ph-5**	2	150	0.5	167	N	172	III
**Ph-6**	N	170	N	172	N	171	III
**Ph-7**	N	170	N	172	N	170	III
**Ph-8**	N	168	N	171	N	172	III
**Ph-9**	N	168	N	170	N	172	III
**Ph-10**	>0.5	164	>1	159	N	171	III
**Tn-1**	N	171	N	168	N	170	III
**Tn-2**	N	168	N	169	N	171	III
**Tn-3**	N	168	>0.5	165	N	168	III
**Tn-4**	1	161	>3	138	1	162	III
**Tn-5**	0.5	167	N	170	N	172	III
**Tn-6**	0.5	167	N	171	N	170	III
**Tn-7**	>1	159	1	161	0.5	167	III
**Tn-8**	0.5	167	N	169	0.5	167	III
**Tn-9**	0.5	167	N	172	N	171	III
**Tn-10**	0.5	167	1	160	0.5	167	III
**Cc-1**	N	171	N	172	N	171	III
**Cc-2**	1	161	>0.5	165	N	170	III
**Cc-3**	>1	159	2	153	1	161	III
**Cc-4**	N	170	N	171	N	168	III
**Cc-5**	N	172	N	172	N	172	III
**Cc-6**	N	172	N	169	N	171	III
**Cc-7**	N	170	>0.5	165	N	170	III
**Cc-8**	N	172	0.5	167	N	172	III
**Cc-9**	>0.5	165	2	153	1	163	III
**Cc-10**	1	162	N	168	N	171	III
**Se-1**	1	160	>0.5	165	1	161	III

**Se-2**	1	162	1	160	0.5	167	III
**Se-3**	>0.5	166	N	170	N	168	III
**Se-4**	>1	159	N	169	>0.5	166	III
**Se-5**	N	170	>3	138	2	154	III
**Se-6**	0.5	167	N	171	N	172	III
**Se-7**	>0.5	166	N	170	N	171	III
**Hw-1**	N	171	>3	138	>3	138	III
**Hw-2**	3	145	N	168	N	172	III
**Hw-3**	N	172	N	169	N	172	III
**Hw-4**	3	144	N	170	N	171	III
**Hw-5**	N	171	>0.5	165	N	171	III
**Hw-6**	N	172	N	171	N	172	III
**Hw-7**	1	163	>1	159	N	171	III
**Hw-8**	>1	159	1	161	N	171	III
**Hw-9**	N	170	N	170	N	170	III
**Hw-10**	>0.5	165	>1	159	N	172	III
**Al-1**	N	172	N	169	N	172	III
**Al-2**	>0.5	165	N	170	N	170	III
**Al-3**	N	171	N	171	N	171	III
**Al-4**	N	172	N	170	N	172	III
**Al-5**	N	170	N	172	N	170	III
**Al-6**	N	172	N	172	N	171	III
**Al-7**	N	171	N	170	N	172	III
**Al-8**	0.5	167	>0.5	165	N	170	III
**Al-9**	N	170	N	171	N	171	III
**Al-10**	N	171	N	171	N	172	III
**Tt-1**	N	168	N	169	N	172	III
**Tt-2**	0.5	167	N	171	N	171	III
**Tt-3**	0.5	167	N	169	N	170	III
**Tt-4**	0.5	167	N	168	N	168	III
**Tt-5**	0.5	167	N	171	N	170	III
**Tt-6**	2	150	>0.5	165	N	172	III

**Tt-7**	N	172	>0.5	164	N	171	III
**Tt-8**	N	170	N	170	N	170	III
**Tt-9**	1	161	N	171	N	172	III
**Tt-10**	0.5	167	N	169	N	171	III
**Ts-1**	0.5	167	1	160	N	168	III
**Ts-2**	0.5	167	1	161	>0.5	165	III
**Ts-3**	0.5	167	N	172	N	168	III
**Ts-4**	0.5	167	N	172	N	172	III
**Ts-5**	0.5	167	N	170	N	171	III
**Ts-6**	N	172	>0.5	164	N	172	III
**Ts-7**	N	171	N	168	0.5	167	III
**Ts-8**	0.5	167	>0.5	164	0.5	167	III
**Ts-9**	N	168	N	170	N	168	III
**Ts-10**	>0.5	166	N	169	N	172	III
**Ac-1**	N	168	N	169	0.5	167	III
**Ac-2**	N	171	N	172	N	172	III
**Ac-3**	0.5	167	N	172	N	170	III
**Ac-4**	N	168	N	171	0.5	167	III
**Ac-5**	>2	148	>3	138	>2	148	III
**Ac-6**	0.5	167	N	170	N	172	III
**Ac-7**	N	168	1	161	N	168	III
**Ac-8**	0.5	167	N	168	N	171	III
**Ac-9**	N	168	N	172	N	170	III
**Ac-10**	N	170	1	160	>0.5	164	III
**Gs-1**	N	168	N	171	N	172	III
**Gs-2**	N	168	N	169	N	171	III
**Gs-3**	N	171	N	170	N	170	III
**Gs-4**	N	168	3	146	N	172	III
**Gs-5**	N	171	0.5	167	N	171	III
**Gs-6**	N	171	1	161	N	170	III
**Gs-7**	N	172	N	168	N	172	III
**Gs-8**	N	171	N	170	N	171	III

**Gs-9**	N	170	N	169	N	168	III
**Gs-10**	>0.5	165	N	168	0.5	167	III
**Cp-1**	N	171	N	172	N	172	III
**Cp-2**	N	171	N	172	N	171	III
**Cp-3**	N	170	>0.5	165	N	168	III
**Cp-4**	N	168	2	152	0.5	167	III
**Cp-5**	N	171	N	171	N	172	III
**Cp-6**	>1	159	>0.5	165	N	171	III
**Cp-7**	0.5	167	N	172	>1	159	III
**Cp-8**	N	172	N	171	N	172	III
**Cp-9**	N	171	N	172	N	171	III
**Cp-10**	3	144	>0.5	165	N	168	III

Hc, healthy control; Ss, proven strongyloidiasis; Gl, giardiasis; Eh, amoebiasis; Bh, blastocystosis; Ov, opisthorchiasis; Fg, fascioliasis; Ph, paragonimiasis; Tn, taeniasis; Cc, cysticercosis; Se, sparganosis; Hw, hookworm infections; Al, ascariasis; Tt, trichuriasis; Ts, trichinellosis; Ac, angiostrongyliasis; Gs, gnathostomiasis; Cp, capillariasis. The intensity cutoff levels for a positive result of the NIE, SsIR, and NIE-SsIR ICT kits were 1, 1, and >0.5, respectively by the naked eye and were < 164, < 164, and < 167, respectively by the in-house strip reader.
